# Aneurysmal Cavity in the Interventricular Septum by the Rupture of the Right Sinus of Valsalva: An Interesting Finding on Cardiac Imaging

**DOI:** 10.7759/cureus.64892

**Published:** 2024-07-19

**Authors:** Gaurav Pandey, Salman Butt, Obaid M Aljassim

**Affiliations:** 1 Cardiothoracic Surgery, Dubai Hospital, Dubai, ARE; 2 Cardiac Surgery, Heart Vascular and Thoracic Institute, Cleveland Clinic Abu Dhabi, Abu Dhabi, ARE

**Keywords:** cardiac magnetic resonance imaging (cmr), cardiac magnetic resonance (cmr), transesophageal echocardiography, aneurysm, interventricular septum, sinus of valsalva

## Abstract

This case report documents a rare congenital anomaly in a 27-year-old man of African descent presenting with exertional chest discomfort and shortness of breath, diagnosed with a ruptured right sinus of Valsalva (RSOV) aneurysm dissecting into the interventricular septum (IVS), creating an aneurysmal cavity. Such occurrences are typically rare, with this type of aneurysm largely manifesting in the right atrium, making its presentation in the IVS without intracardiac communication exceptionally uncommon. Cardiac imaging, including transesophageal echocardiography and cardiac magnetic resonance imaging (CMR), played pivotal roles in visualizing the structural abnormality and planning the subsequent surgical intervention. The patient's treatment included heart failure optimization, followed by surgery to repair the aneurysmal cavity while preserving the native aortic valve. Postoperative challenges included a complete heart block managed by cardiac resynchronization therapy and an intracardiac defibrillator. The report underscores the importance of advanced imaging in diagnosing and managing rare cardiac anomalies, highlighting the aneurysm's unique rupture pattern and location.

## Introduction

We report a case of a 27-year-old male of African origin who presented with intermittent shortness of breath and chest discomfort on exertion for six months.

On cardiac imaging studies, he was found to have a ruptured right sinus of Valsalva (RSOV) aneurysm dissecting into an interventricular septum (IVS), giving the appearance of an aneurysmal cavity inside the heart. This is a rare congenital cardiac anomaly with a variety of presentations, ranging from an accidental finding on routine examination to acute coronary syndrome and sudden cardiac death.

The rupture of a dilated aneurysm of the RSOV into the right atrium is a common presentation. But the same happens in the IVS, and causing an aneurysm within the septum without any intracardiac communication is highly uncommon.

Sinus of Valsalva aneurysm (SVA) has a very low prevalence, with a reported incidence of 0.5%-1.5% in the Western world, 1.2%-4.9% in the Asian population [1-3], and 0.78% of all congenital open heart operations [4]. Aneurysmal dilatation affects the RSOV in 70% of cases, the non-coronary sinus in 25% of cases, and the left sinus of the Valsalva in 5% of cases [5]. Dilated sinuses are always at increased risk for rupture, compression, and sudden decompensation, resulting in heart failure.

The diagnosis of this is an indication of early surgical intervention, as the mean survival period for untreated RSOV patients is one to 3.9 years [6].

## Case presentation

A 27-year-old obese gentleman (BMI: 34 kg/m^2^) presented with acute exacerbations of chronic shortness of breath and exertional chest discomfort.

On examination, he exhibited tachycardia and a wide pulse pressure. A 12-lead ECG showed sinus tachycardia and a left bundle branch block (LBBB). A chest X-ray revealed cardiomegaly with prominent bronchovascular markings.

**Figure 1:**
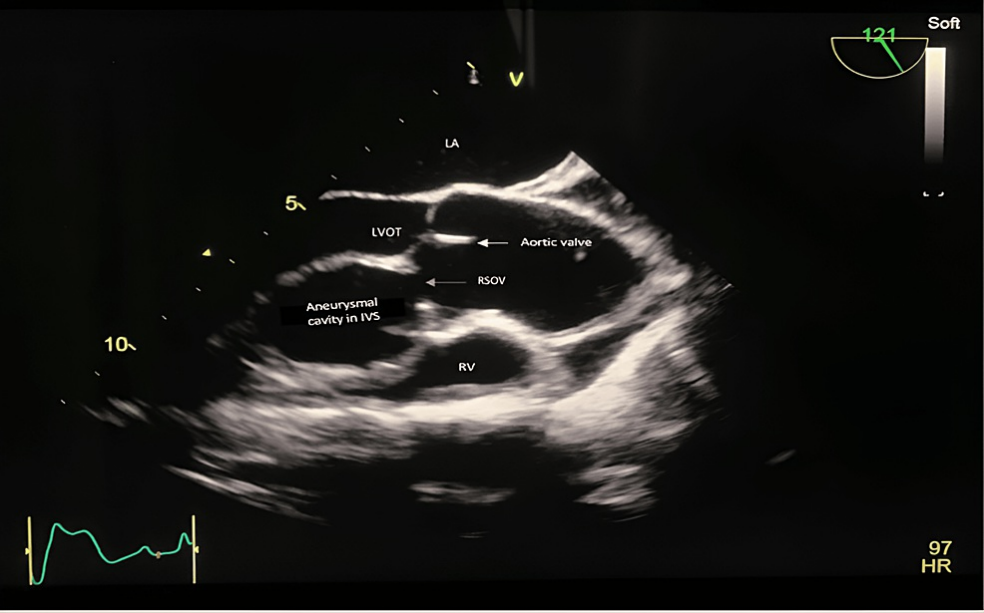
A mid-esophageal long-axis view of the aortic valve demonstrates the dilation and rupture of the right aortic sinus into the interventricular septum, leading to the formation of an aneurysmal cavity within it. LA: left atrium; LVOT:  left ventricular outflow tract; RSOV: right sinus of Valsalva; IVS: interventricular septum; RV: right ventricle

**Figure 2:**
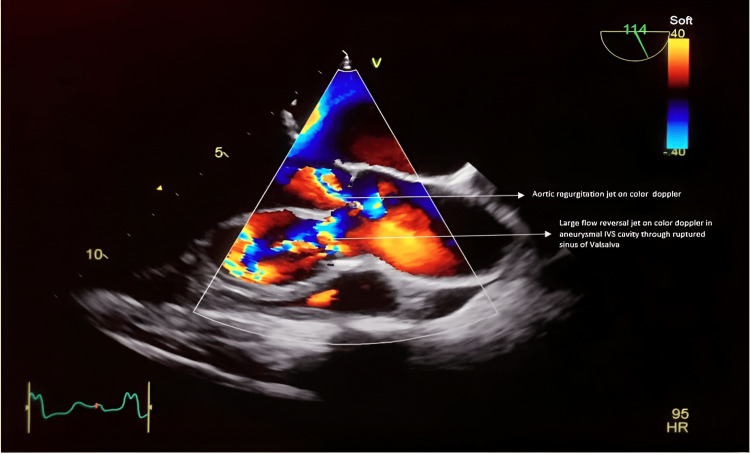
Two-dimensional transesophageal echocardiography imaging on color Doppler highlights regurgitation jets observed in the left ventricular outflow tract and within the aneurysmal cavity in the interventricular septum.

Due to a poor transthoracic echocardiographic window, preoperative transesophageal echocardiography (TEE) was planned, which showed dilation and rupture of the RSOV into the IVS, causing a cavity-like appearance within the IVS (Figures 1, 2, and Videos 1-5). Dilation of the left ventricle with left ventricular end-diastolic diameter (LVEDD) and left ventricular end-systolic diameter (LVESD) was 76 mm and 60 mm, respectively, with severe left ventricular dysfunction and an ejection fraction (EF) of 25%. It also showed moderate aortic incompetence, mild mitral incompetence, and mild pericardial effusion.

**Video 1:** Two-dimensional transesophageal echocardiography imaging reveals a mid-esophageal long-axis view of the aortic valve, featuring color Doppler indicating two regurgitant jets during diastole: one in the left ventricular outflow tract and the other in the dissected aneurysmal cavity within the interventricular septum.

**Video 2:** Two-dimensional transesophageal echocardiography imaging displays a mid-esophageal long-axis view of the aortic valve with a dissected aneurysmal cavity in the interventricular septum and a ruptured sinus of Valsalva.

**Video 3:** Two-dimensional transesophageal echocardiography of the mid-esophageal aortic valve, short-axis view with color Doppler demonstrates rupture of the right coronary sinus and a regurgitant jet during diastole.

**Video 4:** In the two-dimensional transesophageal echocardiogram, the mid-esophageal aortic valve (short-axis view) reveals a ruptured right coronary sinus located at the four o'clock position.

**Video 5:** In the two-dimensional transesophageal echocardiogram (mid-esophageal 4-chamber view), the right atrium, right ventricle, tricuspid valve, and interventricular septum are clearly visible. Within the interventricular septum, a small cavity is noticeable. Additionally, there is a small pericardial effusion present, without any diastolic or systolic collapse.

**Figure 3:**
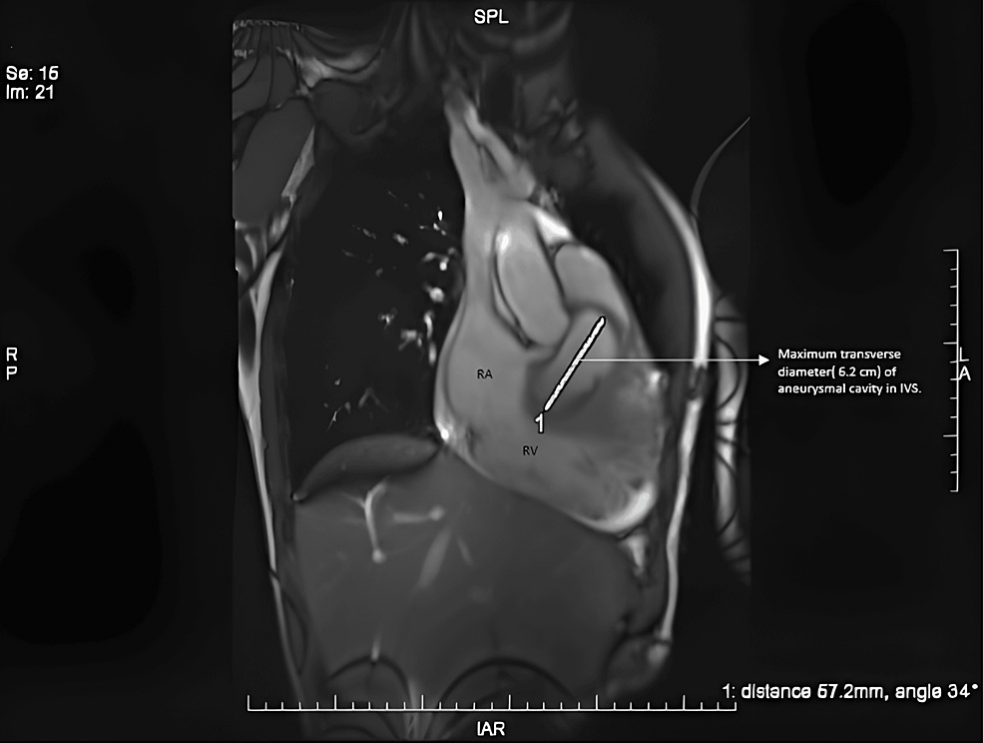
Cardiac MRI depicts the maximum dimension (57.2 mm) of the aneurysmal cavity within the interventricular septum (IVS).

**Figure 4:**
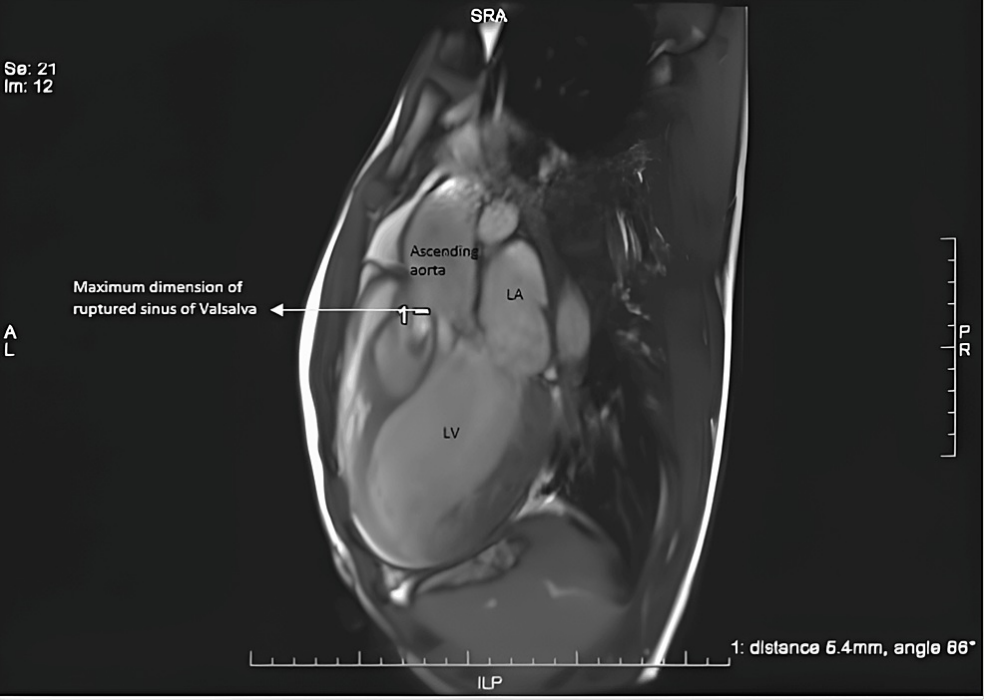
Cardiac MRI represents the dimension of a ruptured opening in the right sinus.

Cardiac magnetic resonance imaging (CMR) (Figures 3-4) was done to rule out any intracardiac shunt or rupture of the aneurysmal cavity and to understand the complete details of the anatomical defect.

The CMR findings confirmed the presence of a large aneurysmal sac within the IVS (dimension of 40 x 57.2 mm) with clear communication with the right coronary sinus. The communication diameter was 5-6 mm, and there was a mural thrombus in the aneurysmal cavity. No evidence of communication between the IVS aneurysmal sac and the left or right ventricle was observed (Qp/Qs-0.95). Left ventricular dilation with severe global systolic dysfunction (EF of approximately 25%) was noted.

After optimization of the patient's heart failure symptoms, the patient underwent surgery for intracardiac repair of the defect while sparing the aortic valve. The patient was shifted to the intensive care unit with epicardial pacing, an intraaortic balloon pump, and inotropic support.

During the recovery phase, the patient developed a complete heart block, for which he was treated with cardiac resynchronization therapy along with the implantation of an intracardiac defibrillator. Eventually, the patient recovered and was discharged from the hospital.

## Discussion

Our patient underwent a successful intracardiac repair of an aneurysmal cavity with native valve-sparing surgery. Due to rupture into the IVS and cavity formation in it, sometimes it is inevitable to develop an irreversible, life-threatening conduction defect post-repair, which might require permanent pacemaker implantation or cardiac resynchronization therapy (CRT), as happened in our case.

A dissecting aneurysm into the IVS is a rare variant of SVA rupture, first reported in 1947 by Warthen [7,8]. It is primarily congenital in origin and usually arises from the right coronary cusp (RCC). Left bundle branch blocks and atrioventricular (AV) blocks can occur, likely due to compression or dissection of the AV bundle within the IVS [9-11].

Computed tomography angiography (CTA) is the investigation of choice [12]. Cine cardiac magnetic resonance imaging, which can evaluate the hemodynamics, identify aortic regurgitation (AR), quantify the shunt or turbulence, and determine the presence of any fistulous tract, is considered the gold standard of investigation [13].

There is little information on the clinical presentation of this entity in Africans. Morais et al. revealed that the main complication of SVA rupture in African patients is usually dissection into the IVS, though they found only four patients with SVA after screening 13,560 patients over a period of six years [14].

## Conclusions

This case report highlights a rare instance of a ruptured RSOV aneurysm leading to an aneurysmal cavity in the IVS. Advanced imaging techniques were critical for diagnosis and surgical planning. Despite postoperative challenges like complete heart block, successful management involved heart failure optimization, cardiac resynchronization therapy, and intracardiac defibrillator implantation. This emphasizes the importance of advanced diagnostic and surgical approaches in handling complex cardiac conditions.
